# Supplementary material for: The effectiveness of group interpersonal synchrony in young autistic adults’ work environment: A mixed methods RCT study protocol
Source: PLoS One. 2024 Jul 31;19(7):e0307956. doi: 10.1371/journal.pone.0307956 (PMC11290619; doi:10.1371/journal.pone.0307956)
Supplement: S1 File — (DOCX) [file pone.0307956.s002.docx]

Research Description

**The effect of group interpersonal synchrony
on work-related soft skills of young adults with ASD**

By: Tamar Dvir ID: 025258351

Supervised by: Dr. Cochavit Elefant & Dr. Tal-Chen Rabinowitch

The prevalence of adults with Autism Spectrum Disorder (ASD) that are able to integrate successfully into the world of work is limited due to difficulties in adapting to social and stressful work environments. The suggested research aims to assess the effect of interpersonal synchrony on work- related soft skills, such as prosocial skills and work-related stress, of young adults who participate in an innovative program “Roim Rachok”, designed to integrate cognitively abled young adults with ASD first into the Israeli army work force, and later into the free market.

Individuals diagnosed with ASD have difficulties with social interaction and communication, as well as repetitive and stereotyped interests (DSM-V, 2013). The prevalence of adults with ASD that are able to successfully integrate into the world of work is limited due to these symptoms, which are often expressed by unsuitable behavior in social situations, difficulty in initiating social interaction, limited interest in forming social relationships, challenges in cooperating with coworkers and difficulty in managing work-related stress (Chevallier et al., 2012; Hedley et al., 2018). The ability to work, according to the International Classification of Functioning, Disability and Health (World Health Organization, 2001) is considered to be a significant factor of individuals’ health, quality of life, and well-being. Therefore, there is a growing interest in forming tailored interventions that will enhance these soft skills among this group of individuals (Gal et al., 2015) in order to more effectively and successfully advance these individuals in work environment.

Interpersonal synchrony, when two or more individuals are sharing body movements or sensations (such as gaze, affect, voice and touch) at the same time, is a key feature of human attachment (Feldman, 2012; Ulmer-Yaniv et al., 2016). It has an important role in mediating cognitive empathy among individuals with ASD (Koehne, Hatri, et al., 2016) and might be used as an effective intervention tool for enhancing emotion inference (Koehne, Behrends, et al., 2016) which is a fundamental skill underlying prosociality. Recently, there is a growing interest in studying the causal influence of interpersonal synchrony on prosocial skills of neurotypical adults in general (Rennung & Göritz, 2016) and specifically in work environments (Göritz & Rennung, 2019). This raises a relatively new interest in studying the same effects on individuals who have difficulties in prosocial skills and who perform poorly in stressful environments, such as individuals with autism who participate in this special “Roim Rachok” program.

**Research Questions**

1. Will the synchronized group intervention have an immediate and long-term effect on participants cooperation and work-related stress?
2. Will this effect be mediated by participants’ achieved social closeness and sense of belonging?
3. Will this effect be affected by participants’ social motivation as reported before the intervention?
4. How will participants perceive the intervention as affecting their work-related soft skills?
5. In what ways will the participants perception of the intervention as affecting their work-related soft skills contribute to a better understanding of the intervention effect?

**Methods**

A mixed methods approach will be applied; collecting and analyzing quantitative and qualitative data. The use of both methods in combination provides a better understanding of the research questions than either method by itself. This study will include the “Explanatory Sequential Design” (Creswell & Clark, 2017) in order to understand more in depth the study’s quantitative results by incorporating the participants’ qualitative perspectives.

**Participants**

The suggested study will be conducted in “Roim Rachok” facilities and will include approximately one hundred 18- to 25-years-old adults diagnosed with ASD who participate in “Roim Rachok’s Program”. Inclusion criteria will include students with an approved ASD diagnosis according to the (DSM-V, 2013). Exclusion criteria will include students with severe sensory impairments such as blindness or deafness and/or severe physical disability. The participants will be recruited from approximately four to five program cycles, each contains between 15-30 students. A new program cycle starts approximately every four months. Therefore, data collection will take between a year and a half to two years.

**Study Design**

The study will be a randomized controlled trial (RCT), which will investigate two conditions of movement-based intervention: synchronous and nonsynchronous, providing each participant with only one type of intervention. The condition groups will be matched according to army related profession, sex, handedness, and belongingness level. Quantitative data collection will be conducted for each participant during three points of time: t0 – Beginning of the “Roim Rachok” course, t1 – End of the course and t2 – Four month after the end of the course. Qualitative data will be collected at a time between t1 – t2 (after the end of the intervention) for 15% of the participants.

**Procedures**

“Roim Rachok” is an innovative program, designed to integrate cognitively abled young adults with ASD first into the Israeli army work force, and later into the free market. As part of the program, before joining the army, the participants take part in a professional three-month course operated by an interdisciplinary team, which includes army officers and instructors, an occupational therapist, a speech therapist and an art therapist. Course contents are composed of two main learning domains: (1) army related profession (e.g., photography interpretation); and (2) integration within the army working environment. A once a week 60 minute movement-based intervention (physical training lesson) is also included, to help the participants cope with the emotional impact of the course requirements. The researcher, who is a professional Dance Movement Therapist working in “Roim Rachok” program, will handle the research approval procedure at the beginning of the “Roim Rachok” course. Each of the course participants will be able to choose whether to participate in this research. Participants decision will not affect their success in the course in any way. The participants will be able to withdraw form this research at any time without this affecting their participation in the program. Participants who have custodian will be required to present the custodian approval as well.

The procedure will contain two phases. At phase one a pilot study will be conducted in order to test the feasibility of a basic 60 minutes movement-based intervention sessions designed by the researcher with the help of a professional physical trainer. The movement-based intervention sessions in the pilot study will be performed by “Roim Rachok” army instructors outside of the “Roim Rachok” course schedule (once in two weeks for the duration of six weeks for each intervention type). The army instructors will be trained by the researcher to apply a condition specific intervention session: synchronous and nonsynchronous movement-based intervention. Each intervention group will contain five participants. At the end of the intervention period the participants will be asked to participate group interview that will be performed by the researcher in order to learn about their perceptions of the intervention goals and effect. This information will help the researcher to better design a full set of intervention protocols for this research. The participants will be asked to volunteer to take part in this pilot study. Their decision will not affect their success in the course in any way.

At phase two the full study (RCT) will be conducted including data collection. The movement-based intervention will be performed by the researcher or by a professional research assistant as part of the “Roim Rachok” course (once a week for the duration of 10 weeks). A graduate student of the “Roim Rachok” program will join as a co-instructor. A condition specific intervention protocol will be applied: synchronous and nonsynchronous movement-based intervention. The protocols will be designed by the researcher with the help of a professional physical trainer. Each protocol will contain 10 physical training sessions, each lasting 60 minutes. The protocols will differ in terms of using synchronous activity forms (e.g. performing same physical exercises together at the same time) vs. using nonsynchronous activity forms (e.g. performing different physical exercises in the form of circuit training). The protocols will not differ in terms of physical exercise type to control for the effect of exercise type on the study’s dependent variables. Each intervention group will contain between seven to fifteen to participants.

**Data Collection**

Quantitative measurements tools

**Background Questionnaires.** The background questionnaires include relevant information such as demographic data (age, gender, religion, country of birth, residential area, number of siblings, birth order and parental education), medical data (IQ, age of ASD diagnosis, comorbidities, use of psychiatric medications, degree of disability in social security, military medical profile) and physiological data (body mass index). Demographic and medical questionnaires are handed out to all “Roim Rachok” trainees during the program selection process, few months before joining the program. During the research approval procedure, the participants will be informed regarding the use of background data for research purposes only.

**Handedness.** handedness will be measured using the Hebrew adaptation of the Edinburgh Handedness questionnaire (Oldfield, 1971), the most common method reported for measuring handedness among individuals with Autism (Preslar et al., 2014).

**Belongingness.** Belongingness will be measured using the Hebrew adaptation of the Need to Belong Scale (Leary et al., 2013). The scale comprises ten items rated on a 5-point scale ranging from “Strongly Disagree” (1) to “Strongly Agree” (5).

**Belongingness level**. Belongingness level (high, medium, low) will be calculated using a cut off of the belongingness measure, so that participants with the 33% highest belongingness score will be assigned a high belongingness level, participants with the 33% lowest belongingness score will be assigned a low belongingness level and the rest will be assigned a medium belongingness level.

**Friendship Closeness.** Friendship Closeness regarding participants in the intervention group will be measured using the Hebrew adaptation of the Friendship Closeness Inventory (FCI; Polimeni, Hardie, & Buzwell, 2002). The FCI includes 49 items that measure closeness of the same-sex friendships and is divided into three distinguishable yet related subscales: Emotional Closeness, Behavioral Closeness, and Cognitive Closeness.

**Social Closeness.** Social Closeness regarding participants in the intervention group will be measured using the Inclusion of Other in Self Scale (IOS) (Aron et al., 1992; Blanchard et al., 1998). This scale includes seven Venn diagram-like pictures where one circle represents the participant and the other circle the represents the entire intervention group. The diagrams, ranging from no overlap to near complete overlap, measure close relationship between self and the group capturing aspects of both feeling close and behaving close.

**Specific Sense of Belonging.** Sense of Belonging regarding participants in the intervention group will be measured using the Hebrew adaptation of the General Sense of Belonging Scale (Malone et al., 2012). The scale includes 12 items that measure sense of belonging (achieved belongingness) rated on a 7-point Likert scale, ranging from “Strongly Disagree” (1) to “Strongly Agree” (7). For the purpose of the present research, the scale was adjusted in that the words “other people” or “others” were replaced by the words “participants in the physical training group”. The original version of the General Sense of Belonging Scale (Malone et al., 2012) will also be used in order to validate the adapted one.

**Work-related stress** Work-related stress will be measured using the Hebrew adaptation of the Irritation Scale (Mohr et al., 2005). The scale comprises eight items, three of which assess cognitive irritation and five of which assess emotional irritation. Items will be rated on a 7-point Likert scale ranging from “Strongly Disagree” (1) to “Strongly Agree” (7).

**Cooperation with co-participants.** Cooperation with co-participants will be measured using the following behavioral group cooperation tasks, both resemble a classic N-person cooperation dilemma as a “collective action problem of a common good” described by Archetti & Scheuring (2012).

(1) Collection task: as described in Jackson, Jong, Bilkey & Whitehouse (2018), in which participants will have to work together to pick up 100 small washers (a flat ring made plastic) scattered across the experimental area. The task will be recorded using one video camera. Cooperation in this task will be operationalized by participants’ effort represented by their step rate (SR). SR will be measured using wearable fitness tracker (Fitbit Inspire 2) that will be attached to participants’ wrist using a special band. Fitbit fitness trackers are commonly used in clinical trials (Henriksen et al., 2018) . In addition, we will normalize SR using physical fitness level by measuring resting heart rate (average heart rate five minutes before the task is performed, while participants are sitting). Fitbit fitness trackers have been found to be highly effective for measuring heart rate especially at rest (Benedetti et al., 2021). Since cooperation in this task will depend on the participants’ physical fitness and might be influenced by group pressure, we will also use a non-physical confidential task to measure a pure voluntary cooperation.

(2) Public good game: as described in (Reddish et al., 2013), in which participants will be told they would be given 30 NIS for which they can contribute some or all to a group investment. The money in the group investment would then be doubled and divided equally among all members of the group. In contrast to the first task, participants contribution will be confidential to avoid group pressure dependency. Cooperation in this task will be operationalized by participants’ amount of money donated to group investment.

Qualitative measurements tools

**Semi- structured interviews.** The semi-structured interviews will explore how participants perceive the movement-based intervention as effecting their working experience during the “Roim Rachok” course. The interviews will be carried out after the end of the course (between t1 and t2) and will be recorded and transcribed. This method consists of a dialogue between the researcher and the participant, guided by a flexible interview protocol and supplemented by follow-up questions and comments (Kvale, 1996).

**References**

Archetti, M., & Scheuring, I. (2012). Review : Game theory of public goods in one-shot social dilemmas without assortment. *Journal of Theoretical Biology*, *299*, 9–20. https://doi.org/10.1016/j.jtbi.2011.06.018

Aron, A., Aron, E. N., & Danny, S. (1992). Inclusion of Other in the Self Scale. *Journal of Personality and Social Psychology*, *63*(4), 596–612. http://dx.doi.org/10.1037/t03963-000

Benedetti, D., Olcese, U., Frumento, P., Bazzani, A., Bruno, S., d’Ascanio, P., Maestri, M., Bonanni, E., & Faraguna, U. (2021). Heart rate detection by Fitbit ChargeHR^TM^: A validation study versus portable polysomnography. *Journal of Sleep Research*, *January*, 1–10. https://doi.org/10.1111/jsr.13346

Blanchard, C., Perreault, S., & Vallerand, R. J. (1998). Participation in team sport: A self-perspective. *International Journal of Sport Psychology*, *29*(4), 289–302.

Chevallier, C., Kohls, G., Troiani, V., Brodkin, E. S., & Schultz, R. T. (2012). The Social Motivation Theory of Autism. *Trends in Cognitive Sciences*, *16*(4), 231–239. https://doi.org/10.1016/j.tics.2012.02.007.The

Creswell, J. W., & Clark, V. L. P. (2017). *Designing and Conducting Mixed Methods Research*. SAGE Publications Ltd.

DSM-V. (2013). *Diagnostic and Statistical Manual of Mental Disorders*. American Psychiatric Association.

Feldman, R. (2012). Oxytocin and social affiliation in humans. *Hormones and Behavior*, *61*(3), 380–391. https://doi.org/10.1016/j.yhbeh.2012.01.008

Gal, E., Selanikyo, E., Erez, A. B. H., & Katz, N. (2015). Integration in the vocational world:How does it affect quality of life and subjective well-being of young adults with ASD. *International Journal of Environmental Research and Public Health*, *12*(9), 10820–10832. https://doi.org/10.3390/ijerph120910820

Göritz, A. S., & Rennung, M. (2019). Interpersonal synchrony increases social cohesion, reduces work-related stress and prevents sickdays: a longitudinal field experiment. *Gruppe. Interaktion. Organisation. Zeitschrift Fur Angewandte Organisationspsychologie*, *50*(1), 83–94. https://doi.org/10.1007/s11612-019-00450-8

Hedley, D., Cai, R., Uljarevic, M., Wilmot, M., Spoor, J. R., Richdale, A., & Dissanayake, C. (2018). Transition to work: Perspectives from the autism spectrum. *Autism*, *22*(5), 528–541. https://doi.org/10.1177/1362361316687697

Henriksen, A., Mikalsen, M. H., Woldaregay, A. Z., Muzny, M., Hartvigsen, G., Hopstock, L. A., & Grimsgaard, S. (2018). Using fitness trackers and smartwatches to measure physical activity in research: Analysis of consumer wrist-worn wearables. *Journal of Medical Internet Research*, *20*(3). https://doi.org/10.2196/jmir.9157

Jackson, J. C., Jong, J., Bilkey, D., & Whitehouse, H. (2018). Synchrony and Physiological Arousal Increase Cohesion and Cooperation in Large Naturalistic Groups. *Scientific Reports*, *8*(1), 1–8. https://doi.org/10.1038/s41598-017-18023-4

Koehne, S., Behrends, A., Fairhurst, M. T., & Dziobek, I. (2016). Fostering Social Cognition through an Imitation- and Synchronization-Based Dance/Movement Intervention in Adults with Autism Spectrum Disorder: A Controlled Proof-of-Concept Study. *Psychotherapy and Psychosomatics*, *85*(1), 27–35. https://doi.org/10.1159/000441111

Koehne, S., Hatri, A., Cacioppo, J. T., & Dziobek, I. (2016). Perceived interpersonal synchrony increases empathy: Insights from autism spectrum disorder. *Cognition*, *146*, 8–15. https://doi.org/10.1016/j.cognition.2015.09.007

Kvale, S. (1996). InterViews. An introduction to qualitative research writing. In *The American Journal of Evaluation* (Vol. 19, Issue 2). https://doi.org/10.1016/s1098-2140(99)80208-2

Leary, M. R., Kelly, K. M., Cottrell, C. A., & Schreindorfer, L. S. (2013). Construct validity of the need to belong scale: Mapping the nomological network. *Journal of Personality Assessment*, *95*(6), 610–624. https://doi.org/10.1080/00223891.2013.819511

Malone, G. P., Pillow, D. R., & Osman, A. (2012). The General Belongingness Scale (GBS): Assessing achieved belongingness. *Personality and Individual Differences*, *52*(3), 311–316. https://doi.org/10.1016/j.paid.2011.10.027

Mohr, G., Rigotti, T., & Müller, A. (2005). Irritation - Ein Instrument zur Erfassung psychischer Beanspruchung im Arbeitskontext. Skalen- und Itemparameter aus 15 Studien. *Zeitschrift Fur Arbeits- Und Organisationspsychologie*, *49*(1), 44–48. https://doi.org/10.1026/0932-4089.49.1.44

Oldfield, R. C. (1971). The assessment and analysis of handedness: The Edinburgh inventory. *Neuropsychologia*, *9*(1), 97–113. https://doi.org/10.1016/0028-3932(71)90067-4

Polimeni, A., Hardie, E., & Buzwell, S. (2002). Friendship closeness inventory: Development and psychometric evaluation. *Psychological Reports*, *91*(1), 142–152.

Preslar, J., Kushner, H. I., Marino, L., & Pearce, B. (2014). Autism, lateralisation, and handedness: A review of the literature and meta-analysis. *Laterality*, *19*(1), 64–95. https://doi.org/10.1080/1357650X.2013.772621

Reddish, P., Fischer, R., & Bulbulia, J. (2013). Let’s Dance Together : Synchrony, Shared Intentionality and Cooperation. *PloS One*, *8*(8). https://doi.org/10.1371/journal.pone.0071182

Rennung, M., & Göritz, A. S. (2016). Prosocial consequences of interpersonal synchrony: A Meta-Analysis. *Zeitschrift Fur Psychologie / Journal of Psychology*, *224*(3), 168–189. https://doi.org/10.1027/2151-2604/a000252

Ulmer-Yaniv, A., Avitsur, R., Kanat-Maymon, Y., Schneiderman, I., Zagoory-Sharon, O., & Feldman, R. (2016). Affiliation, reward, and immune biomarkers coalesce to support social synchrony during periods of bond formation in humans. *Brain, Behavior, and Immunity*, *56*, 130–139. https://doi.org/10.1016/j.bbi.2016.02.017
