# Supplementary material for: The effectiveness of group interpersonal synchrony in young autistic adults’ work environment: A mixed methods RCT study protocol
Source: PLoS One. 2024 Jul 31;19(7):e0307956. doi: 10.1371/journal.pone.0307956 (PMC11290619; doi:10.1371/journal.pone.0307956)
Supplement: Attachment — Submitted filename: Response to Reviewers.docx [file pone.0307956.s003.docx]

PONE-D-23-06520

**The effectiveness of group interpersonal synchrony in young autistic adults’ work environment: A mixed methods RCT study protocol**

PLOS ONE

Dear Editor,

Thank you for the most helpful review. We have addressed all the reviewer’s comments (in blue) with a detailed response (in black), followed by a quote from the revised manuscript and an exact page and line number. We feel the manuscript has greatly benefited from this revision and are grateful for the constructive comments from the reviewer.

Sincerely,

Tamar Dvir

Tamar Dvir, Tal-Chen Rabinowitch, Cochavit Elefant

University of Haifa, Faculty of Social Welfare & Health Sciences, School of Creative Arts Therapies, 199 Aba Khoushy Ave., Mount Carmel, Haifa 3498838, Israel

**Reviewer #1 Evaluation:**

The abstract reads as if it is a final paper, when it only becomes obvious that it is not by the absence of a results section. This should have been made clearer at the outset.

Thank you for this important comment. We revised the abstract to clearly state that this is a study protocol (on page 2 lines 19-20):

This study protocol outlines research that aims to assess the effects of group interpersonal synchrony on prosociality and work-related stress of young autistic adults in their work environment.

Also, it would have been good to read about some preliminary results from the pilot study.

Thank you for suggesting to include preliminary results from the pilot. We revised the Study status section to clearly state that no data was collected during the assessment of the intervention protocol (on page 26 lines 514-518), therefore there are no preliminary results:

A feasibility assessment [72] of the intervention protocol was conducted to fine-tune the intervention protocol by documenting the experiences of the participants in terms of the goals of each of the intervention conditions (see above in Procedure). Participants' allocation to intervention conditions during the feasibility assessment was not randomized or controlled and no data was collected.

The work plan looks reasonable, with a decent procedure and interesting question., There is a good number of participants (N=60).

Thank you for your helpful and encouraging feedback.

I have a number of minor questions, however:
Why aren't all participants interviewed?
Thank you for this question. We revised the Qualitative data collection section to state that interviews will be conducted till no new themes or insights emerge from additional interviews (on page 23 line 461-462):

Qualitative data will be collected from the participants till data saturation is achieved, using semi-structured interviews.

The five studies that are referred to on p25 -31 need to be more clearly stated to remind the readers that they refer to the original objectives of p15. i.e. a clearer link.

Thank you for this important comment. We revised the Data analysis section to refer to the original objectives (on page 25 line 489-490):

To answer the five study objectives (see above in Introduction) the following data analysis will be conducted:

The use of Fitbits to monitor step counting is an interesting approach. It would be helpful to elaborate on exactly how step counting is related to cooperation. In that same study, I am also curious as to whether it is cooperation vs competition that might be the driving force. (There is related work using similar wearables to measure social behaviour in autism, that it might be helpful to look into.)

Thank you for these important comments. We revised the Methods section to elaborate on the driving force in this specific cooperation task and to link step counting to cooperation via calculating step rate, representing the participant’s effort (on page 22-23 line 433-446):

In this specific task, the act of gathering washers embodies the concept of the common good. Participants must collect all washers and place them in a small basket before returning to their seats. However, the temptation to free ride, relying on others' efforts, illustrates the collective action problem. While it benefits both individuals and the group to gather the washers, the impact of any single person's contribution is minimal and difficult to discern. This dynamic creates an incentive to conserve energy by abstaining from action and allowing others to undertake the task. Participants must work together toward a common goal (collecting all washers in the shortest time), therefore there is likely no incentive to compete. As in previous social loafing paradigms [67], cooperation on this task will be operationalized by participants’ effort to overcome the incentive to conserve energy, represented by their step rate (number of steps per second). The number of steps will be measured using a wearable fitness tracker (Fitbit Inspire 2) that will be attached to the participants’ wrists using a special band.

Early in the manuscript the phrase, "who developed ASD during her second year of life", might be problematic for some – it makes various assumptions about the causes of autism. Perhaps, “who were diagnosed as ASD” might be more appropriate.

Thank you for this important comment. We revised the Introduction section as follows (on page 4 line 61):

Asynchronous social behavior in autism (e.g., reduced eye contact, absence of well-timed co-regulation, no coherent engagement of mutual attention, no anticipation and no emotional build up in shared interactions) was first identified in a micro-analytic study of videos of an 11-month old infant who was diagnosed with ASD during her second year of life
