# Supplementary material for: The effectiveness of group interpersonal synchrony in young autistic adults’ work environment: A mixed methods RCT study protocol
Source: PLoS One. 2024 Jul 31;19(7):e0307956. doi: 10.1371/journal.pone.0307956 (PMC11290619; doi:10.1371/journal.pone.0307956)
Supplement: Attachment — Submitted filename: Response to Reviewers.docx [file pone.0307956.s004.docx]

PONE-D-23-06520

**The effectiveness of group interpersonal synchrony in young autistic adults’ work environment: A mixed methods RCT study protocol**

PLOS ONE

Dear Editor,

Thank you for the most helpful review. We have addressed all the reviewer’s comments (in blue) with a detailed response (in black), followed by a quote from the revised manuscript and an exact page and line number. We feel the manuscript has greatly benefited from this second revision and are grateful for the constructive comments from the reviewer.

Sincerely,

Tamar Dvir

Tamar Dvir, Tal-Chen Rabinowitch, Cochavit Elefant

University of Haifa, Faculty of Social Welfare & Health Sciences, School of Creative Arts Therapies, 199 Aba Khoushy Ave., Mount Carmel, Haifa 3498838, Israel

**Reviewer #2 Evaluation:**

What is the “mixed methods approach”?
We revised the abstract to clearly state that a convergent mixed methods design will be applied (on page 9 line 189-190):

A convergent mixed methods design will be applied, where quantitative and qualitative data are collected and analyzed in parallel.

Need to specify primary aims (confirmatory?) and secondary aims (exploratory?).

Thank you for this important suggestion. We revised the abstract to specify one primary objective and four secondary objectives (on page 9 lines 178-186):

The primary objective is to determine whether a synchronized group intervention will have an immediate and/or long-term positive effect on participants’ prosociality and work-related stress. The secondary objectives are to determine: (1) Whether a synchronized group intervention will have an immediate and/or long-term positive effect on participants’ social closeness and sense of belonging, (2) Whether this effect will be influenced by participants’ need to belong as reported before the intervention, (3) How participants perceive the intervention as affecting their prosociality and work-related stress, and (4) In what ways the participants’ perception will contribute to a better understanding of the intervention effect.

The small sample size will not have sufficient power for so many hypothesis testings. The authors mentioned that the sample size will not support mediating or moderating analysis, then these analyses may not be performed.

Thank you for this important comment. Since we mentioned that the sample size might not support mediating analysis, we simplified the theoretical model and data analysis (on the Introduction section pages 7-8 lines 145-171, Figure 2 page 9 line 173, and the Quantitative data analysis section pages 25-26 lines 503-515).

Sample size calculation: F=0.2 needs to be justified.

We revised the abstract to justify the selection of the effect size defined as f=0.25 for sample size calculation that supports the moderating model (on pages 11-12 lines 237-244):

A meta-analysis on prosocial consequences of interpersonal synchrony among neurotypical adults found a medium-sized effect of interpersonal synchrony on prosociality with regard to both attitudes and behavior towards individuals or groups involved in the same intervention [28]. Therefore, an effect defined as d = 0.25 may be expected [50]. An a-priori power analysis using the G*Power computer program [49] indicated that a total sample size of 40 participants would be needed to detect medium-sized effects defined as f=0.25, with 80% power and alpha at .05, using a repeated measure, within-between interaction ANOVA with four groups and three measurements.

Specify primary endpoint and secondary endpoints.

We revised the abstract to specify primary and secondary outcomes (Table 2 on page 20 line 383):

**Table 2. Study outcomes**

**Outcomes**

**Primary Outcomes**

Work-related stress

Non-confidential behavioral cooperation

Confidential behavioral cooperation

**Secondary Outcomes**

Social closeness

Friendship closeness

Sense of belonging

Line 261: “between-subject” can be omitted.

We omitted the “between-subject” (on Page 12 line 262).

P values needs to adjusted for multiple tests.

Thank you for this important comment. We revised the abstract to state that we will use a statistical procedure to adjust P values for multiple tests (on page 25 lines 498-499):

To control the family-wise error rate when performing multiple hypothesis tests, Bonferroni Step-Down (Holm) will be applied.
